# Orientation relationship of eutectoid FeAl and FeAl_2_

**DOI:** 10.1107/S1600576716000911

**Published:** 2016-02-24

**Authors:** A. Scherf, A. Kauffmann, S. Kauffmann-Weiss, T. Scherer, X. Li, F. Stein, M. Heilmaier

**Affiliations:** a Institute of Applied Materials (IAM-WK), Karlsruhe Institute of Technology (KIT), Karlsruhe, Germany; b Institute for Technical Physics (ITEP), Karlsruhe Institute of Technology (KIT), Karlsruhe, Germany; c Institute of Nanotechnology (INT), Karlsruhe Institute of Technology (KIT), Karlsruhe, Germany; d Karlsruhe Nano Micro Facility (KNMF), Karlsruhe Institute of Technology (KIT), Hermann-von-Helmholtz-Platz 1, Eggenstein-Leopoldshafen, 76344, Germany; e Structure and Nano-/Micromechanics of Materials, Max-Planck-Institut für Eisenforschung, Düsseldorf, Germany

**Keywords:** iron aluminides, eutectoid decomposition, electron backscatter diffraction, interfaces

## Abstract

Fe–Al alloys in the aluminium range of 55–65 at.% exhibit a lamellar microstructure of B2-ordered FeAl and triclinic FeAl_2_, which is caused by a eutectoid decomposition of the high-temperature Fe_5_Al_8_ phase, the so-called ∊ phase. The orientation relationship of FeAl and FeAl_2_ has previously been studied by Bastin *et al.* [*J. Cryst. Growth* (1978 ▸), **43**, 745] and Hirata *et al.* [*Philos. Mag. Lett.* (2008 ▸), **88**, 491]. Since both results are based on different crystallographic data regarding FeAl_2_, the data are re-evaluated with respect to a recent re-determination of the FeAl_2_ phase provided by Chumak *et al.* [*Acta Cryst.* (2010 ▸), C**66**, i87]. It is found that both orientation relationships match subsequent to a rotation operation of 180° about a 〈112〉 crystallographic axis of FeAl or by applying the inversion symmetry of the FeAl_2_ crystal structure as suggested by the Chumak data set. Experimental evidence for the validity of the previously determined orientation relationships was found in as-cast fully lamellar material (random texture) as well as directionally solidified material (∼〈110〉_FeAl_ || solidification direction) by means of orientation imaging microscopy and global texture measurements. In addition, a preferential interface between FeAl and FeAl_2_ was identified by means of trace analyses using cross sectioning with a focused ion beam. On the basis of these habit planes the orientation relationship between the two phases can be described by (01)_FeAl_ || (114) and [111]_FeAl_ || [10]. There is no evidence for twinning within FeAl lamellae or alternating orientations of FeAl lamellae. Based on the determined orientation and interface data, an atomistic model of the structure relationship of Fe_5_Al_8_, FeAl and FeAl_2_ in the vicinity of the eutectoid decomposition is derived. This model is analysed with respect to the strain which has to be accommodated at the interface of FeAl and FeAl_2_.

## Abstract 2

The orientation relationship and interface plane of eutectoid FeAl and FeAl_2_ lamellae are investigated in detail and a crystallographic model is proposed.

## 1. Introduction   

Iron aluminides are potential candidates for structural applications at high temperatures due to their outstanding oxidation resistance, low density and low material cost. However, mechanical properties such as moderate room-temperature ductility and a sharp drop in strength at 873 K limit their usability. Moreover, above 40 at.% Al the brittle-to-ductile transition temperature increases to about 1023 K (Risanti *et al.*, 2005 ▸). However, in the aluminium range of 55–65 at.% the high-temperature ∊ phase Fe_5_Al_8_ (space group 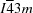, No. 217, Pearson symbol *cI*52) (Stein *et al.*, 2010 ▸) decomposes by a solid-state eutectoid reaction into B2-ordered FeAl (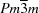, No. 221, *cP*2) (Stein *et al.*, 2010 ▸) and triclinic FeAl_2_ (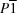, No. 2, *aP*19) (Chumak *et al.*, 2010 ▸). This reaction results in a lamellar microstructure, which may yield an improvement of the mechanical properties similar to what is commonly known from TiAl-based alloys (Huang, 1992 ▸). In the case of TiAl-based alloys, it has been shown that the mechanical properties strongly depend on colony size, lamellar spacing and lamellar orientation with respect to the loading axis (Yamaguchi *et al.*, 2000 ▸; Fujiwara *et al.*, 1990 ▸). The influence of the latter was evaluated using single crystals consisting of single colonies (Fujiwara *et al.*, 1990 ▸; Inui *et al.*, 1992 ▸). By applying the knowledge about this anisotropic behaviour it is possible to optimize the microstructural development and, therefore, mechanical properties – for example by a suitable directional solidification process. However, the control of the eutectoid microstructure by directional solidification in the case of Fe–Al needs a detailed characterization of the relationship between the decomposing ∊ phase and the resulting phases FeAl and FeAl_2_.

In the present study, the orientation relationship, as determined previously by Bastin *et al.* (1978 ▸) and Hirata *et al.* (2008 ▸), will be confirmed and the supposed differences will be examined. In addition, the interface of the FeAl and FeAl_2_ lamellae is investigated by means of local orientation determination and cross sectioning. In contrast to the investigation of Bastin *et al.* (1978 ▸), a distinct preferential interface can be identified. By applying the experimental data, the atomistic details of the relationship of the involved crystal structures are discussed. A possible orientation and interface relationship of FeAl and FeAl_2_ to the high-temperature ∊ phase during the eutectoid reaction is proposed.

## 2. Experimental   

Two rods of an alloy with a nominal composition of 60.5 at.% Al were produced by arc melting and subsequent casting into a copper mould with dimensions of Ø 13.5 × 170 mm. One of the rods was directionally solidified using additional zone melting. The velocity was 18 mm h^−1^ at a temperature gradient of roughly 30 K mm^−1^.

In order to investigate the orientation relationship of FeAl and FeAl_2_, orientation imaging microscopy by electron backscatter diffraction (EBSD) and texture analysis by X-ray diffraction (XRD) were carried out. EBSD was performed utilizing a Zeiss Auriga cross-beam scanning electron microscope (SEM) operated at 20 kV (with 120 µm aperture) and equipped with an EDAX DigiView EBSD detector. The orientation maps of 100 × 100 µm were obtained by indexing patterns of 686 × 686 pixels (maximum resolution) with eight to ten bands at a point rate of about 25 s^−1^ and a step size of 0.1 µm. The fraction of indexed points is above 99.8%. For the maps on as-cast material and on directionally solidified material 70 and 73%, respectively, of the points were recorded with confidence indices (CIs) above 0.1. The contribution of low CIs is mainly attributed to regions with overlapping patterns of both phases near the phase boundaries. Thus, small lamella spacing and low inclination with respect to the surface locally result in a higher fraction of low CIs. Nevertheless, for all investigated regions reliable information for the lamella orientations was observed. The maps in Figs. 4 and 5 below are shown without any cleaning operation. Cross sectioning was performed using the focused Ga ion beam (FIB) of the Auriga microscope at 30 kV and 16 nA. XRD texture measurements were carried out on a Bruker D8 diffractometer equipped with an Eulerian cradle. The Cu X-ray tube was operated at 40 kV and 40 mA. In order to determine the exact Bragg positions on the directionally solidified material, measurements with ω offset were initially performed. Using ω offset, peak positions of inclined (with respect to the sample surface) sets of crystallographic planes can be analysed. For this kind of measurement a motorized slit of 0.7 mm and a 2.5° axial Soller slit were used at the source as well as two motorized slits of 9 mm, a 2.5° axial Soller slit and an Ni filter at the Lynxeye-XE detector. For texture measurements a Göbel mirror, circular slit of 1 mm in diameter and collimator of 1 mm were used at the source as well as two motorized slits of 9 mm at the Lynxeye-XE in 0D mode with three channels. The pole figures were determined between 0 and 360° in φ as well as 0–80° in Ψ with steps of 4°. The defocusing-induced intensity decrease with increasing tilting angle was estimated by accompanying measurements on powder samples of different materials. For both kinds of experiments, cross sections were prepared by a standard metallographic procedure including grinding up to a grid of P4000 and subsequent polishing with 1 µm diamond suspension as well as oxide polishing suspension OP-S provided by Buehler.

## 3. Results and discussion   

### 3.1. Crystallographic data from literature – revisited   

The orientation relationship of FeAl and FeAl_2_ was previously investigated by Bastin *et al.* (1978 ▸) and Hirata *et al.* (2008 ▸) using the Weissenberg technique and transmission electron microscopy, respectively. Thereby, Bastin *et al.* revealed 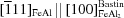 and 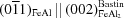 based on a pseudo-monoclinic description of the FeAl_2_ crystal structure in directionally solidified, fully lamellar material (Livingston, 1973 ▸).^1^ Despite the fact that Bastin *et al.* investigated fully lamellar material, they were not able to identify preferential interface planes between the two structures. Hirata *et al.* (2008 ▸) determined 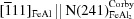 and 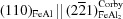 using crystallographic data (triclinic description) for FeAl_2_ provided by Corby & Black (1973 ▸). Note that the angle between 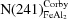 and 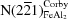 is 91.26° whereas 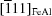 and 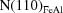 are perpendicular. This issue is addressed in §3.2. In their approach, an Al-lean alloy with 55 at.% Al, exhibiting a B2 matrix with FeAl_2_ particles, was used. Because particles were analysed, preferential interfaces were not obtained. Since it cannot easily be rationalized whether the two relationships are similar or not, a comparison of the two data sets was carried out first. For this purpose, suitable transformations of the crystallographic data were examined with respect to a recent re-determination described as follows.

**Figure 1:**
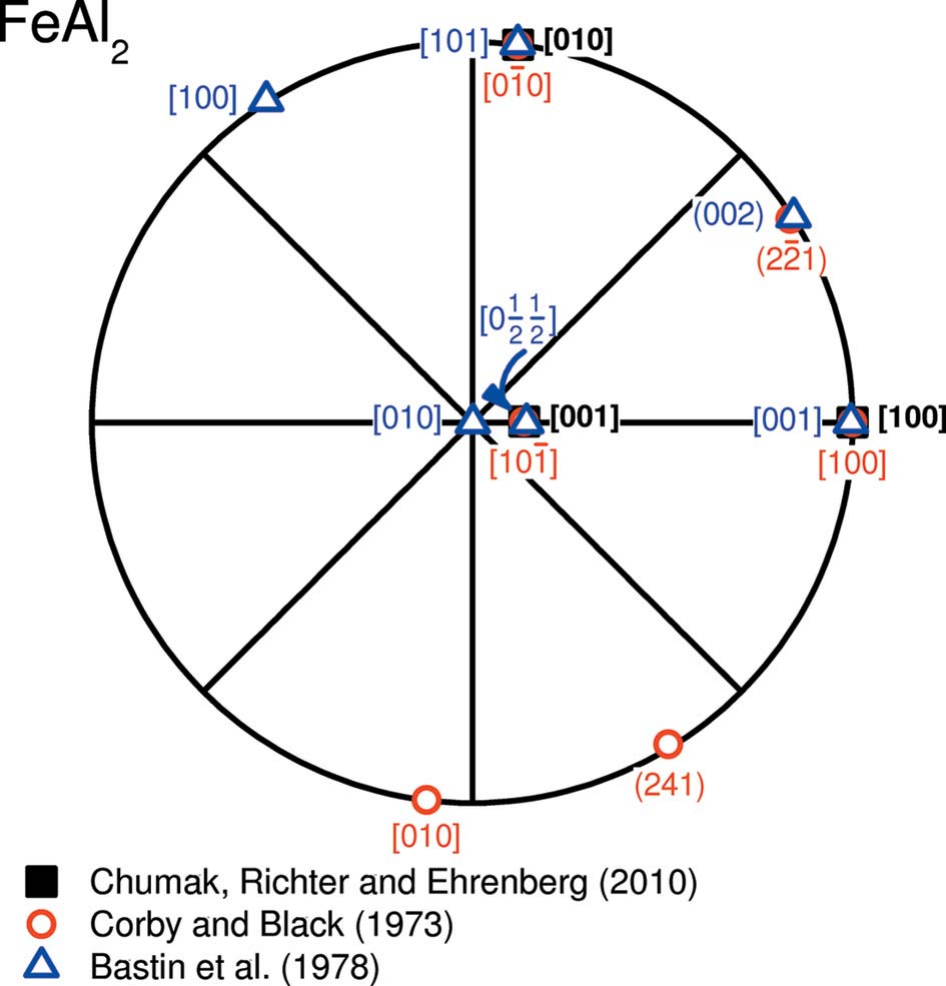
Stereographic projection of the transformed basis vectors according to the different descriptions of the FeAl_2_ phase with respect to the unit cell reported by Chumak *et al.* (2010 ▸).

Crystallographic data by Chumak *et al.* (2010 ▸) are used as the reference data for FeAl_2_. The lattice parameters are summarized in Table 1 ▸. For comparison, the original crystallographic data, as used by Bastin *et al.* (1978 ▸) and Hirata *et al.* (2008 ▸), are included in Table 1 ▸ as well. In the case of Corby & Black (1973 ▸), a suitable transformation is given by the Inorganic Crystal Structure Database (ICSD, collection code 150477) as **a**′ = **a**, **b**′ = −**b** and **c**′ = **a** − **c**. The transformation of the data, determined by Bastin *et al.* (1978 ▸), was derived to be **a**′ = **c**, **b**′ = **a** + **c** and **c**′ = 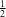
(**b** + **c**). For the description of the crystal structure in a pseudo-monoclinic framework, Bastin *et al.* (1978 ▸) used a non-primitive unit cell exhibiting twice the volume of the primitive unit cell evaluated by Chumak *et al.* (2010 ▸). In order to visualize the derived transformations, the basis vectors transformed accordingly are presented in stereographic projection in Fig. 1 ▸ with respect to the unit cell of Chumak *et al.* (2010 ▸).

The congruence of 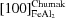 ≡ 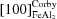 ≡ 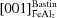, 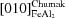 ≡ 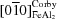 ≡ 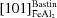 as well as of 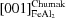 ≡ 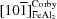 ≡ 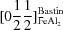 in the stereographic projection in Fig. 1 ▸ illustrates that the descriptions of FeAl_2_ are similar and can be transformed appropriately. In order to compare the previously determined orientation relationships, transformed and reoriented data for the triclinic FeAl_2_ phase will be used in the following.

**Figure 2:**
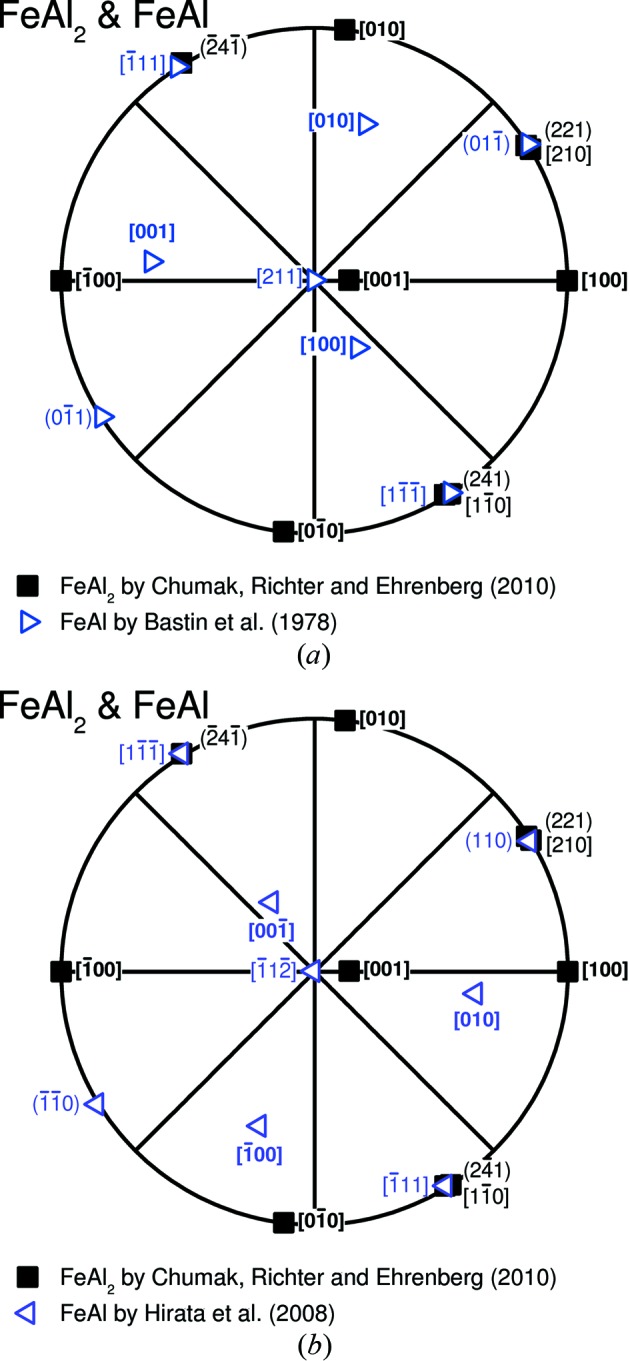
Stereographic projection of the orientation relationship between FeAl and FeAl_2_ according to (*a*) Bastin *et al.* with 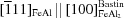 and 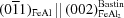 [in terms of the Chumak data (Chumak *et al.*, 2010 ▸): 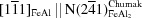 and 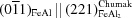], (*b*) Hirata *et al.* with 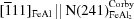 and 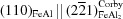 [in terms of the Chumak data (Chumak *et al.*, 2010 ▸): 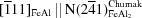 and 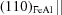 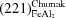]. For the pole projection of lattice planes, the normal vector of the planes is used.

The stereographic projections presenting the orientation relationship between FeAl and FeAl_2_ according to Bastin *et al.* (1978 ▸) and Hirata *et al.* (2008 ▸) are shown in Fig. 2 ▸. This clearly reveals that the two orientation relationships are not necessarily identical. It is possible to achieve congruence of both data sets by a rotation operation of 180° about a 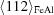 direction in the centres of Figs. 2 ▸(*a*) and 2 ▸(*b*) or by applying the inversion symmetry of the FeAl_2_ phase, which is assumed by the crystallographic data in Chumak *et al.* (2010 ▸). In the latter case, both orientation relationships describe the same situation. In contrast, a 180° rotation about a 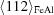 axis corresponds to the common twin system 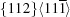 of body-centred cubic metals (Christian & Mahajan, 1995 ▸) as well as of the ordered structures derived from that (Cahn & Collect, 1961 ▸). In principle, the two twin-related orientations of FeAl can be caused by either twin boundaries within a single FeAl lamella which form during the transformation of the ∊ phase to FeAl or growth of two different variants of FeAl separated by the FeAl_2_ phase. These two possibilities can be examined with automated orientation imaging microscopy using EBSD as has been done in this study. The occurrence of inversion symmetry of FeAl_2_ or inversion twinning within FeAl_2_ cannot be investigated by the applied methods.

### 3.2. As-cast material   

**Figure 3:**
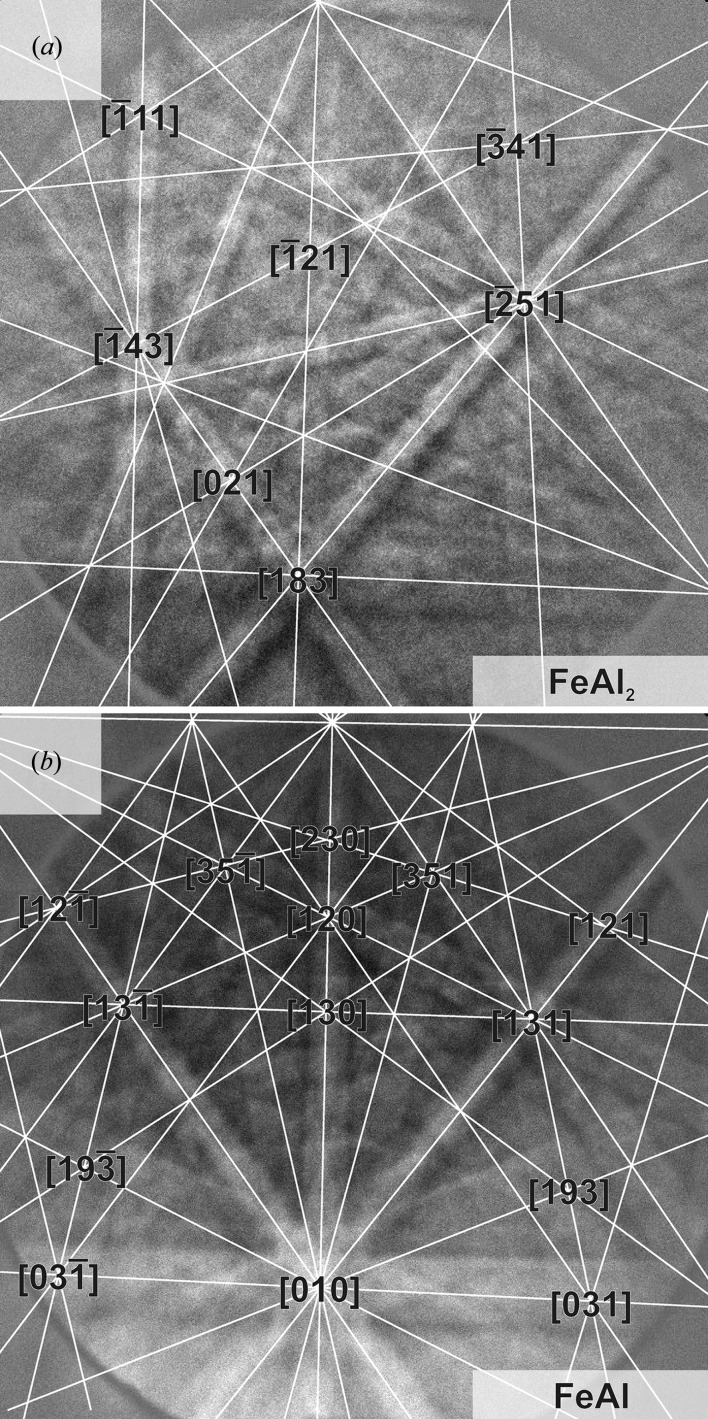
Electron backscatter patterns of (*a*) FeAl_2_ and (*b*) FeAl.

Figs. 3 ▸(*a*) and 3 ▸(*b*) show examples of the obtained electron backscatter patterns for the later orientation determination of FeAl_2_ and FeAl, respectively. Despite a low number of zone axes in the case of FeAl_2_, a robust assignment of zone axes was possible in order to determine phase and orientation within the probed sample volume.

**Figure 4:**
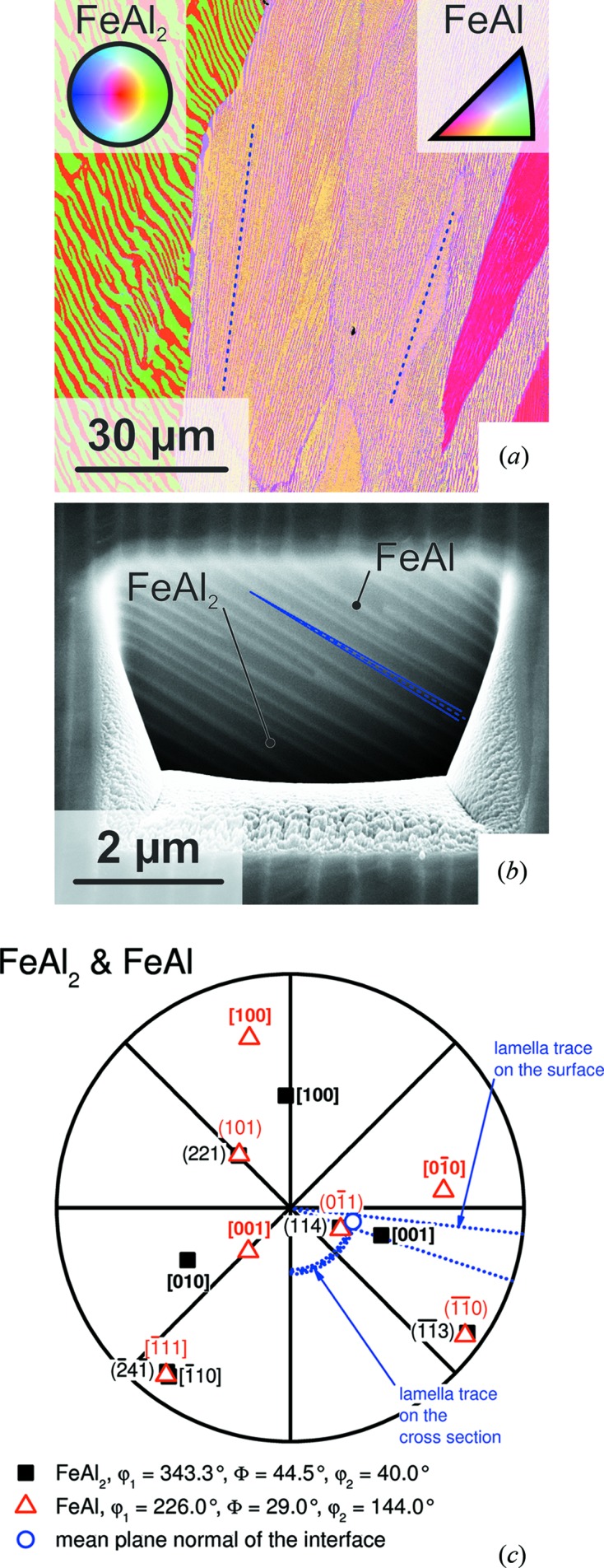
(*a*) Orientation mapping according to the inverse pole figure of the surface normal on a cross section of the arc-melted button (see insets; centre of the IPF of FeAl_2_ corresponds to 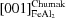, standard triangle of FeAl encloses 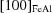, 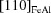 and 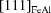). Blue lines indicate minima and maxima of the lamella trace rotation with respect to the horizontal edge of the colony in the centre of the image. (*b*) An example of a SEM micrograph on a cross section prepared by FIB (secondary electron contrast, tilt-corrected). The blue dashed line indicates the mean of the lamella trace inclination with respect to the sample surface obtained from several cross sections. The standard deviation is highlighted by dotted lines. (*c*) Stereographic projection of the determined orientations of the colony in the centre of (*a*). Blue lines indicate rotation and inclination of the lamella traces according to (*a*) and (*b*). The complete set of stereographic projections for all colonies in (*a*) is provided as supporting information. For the pole projection of lattice planes, the normal vector of the planes is used.

The orientation mapping [colour code according to the inverse pole figure (IPF) of the sample surface normal, see insets and description of Fig. 4 ▸(*a*)] of the as-cast state in Fig. 4 ▸(*a*) shows four different colonies of varying orientation. The volume fractions of the phases are approximately 40 and 60 vol.% for FeAl and FeAl_2_, respectively. This is higher than the theoretical evaluation which leads to 28 and 72 vol.% based on the phase diagram and the crystallographic data. The shift of the relative proportions might be attributed to the high cooling rate in the water-cooled copper mould. The rotation of the lamellar traces on the surface for the colony in the centre of Fig. 4 ▸(*a*) is marked by blue dashed lines indicating maximum deviation from the mean rotation. The stereographic projection in Fig. 4 ▸(*c*) illustrates the determined orientation relationship of the two phases within the colony in the centre of the image [the complete set of stereographic projections for all colonies in Fig. 4 ▸(*a*) is provided as supporting information]. In accordance with the previous investigations, 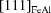 is almost parallel to 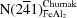 and 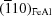 is parallel to 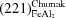 in all cases. Nevertheless, as mentioned before the angle between 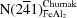 and 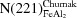 remains 91.26°, indicating that 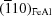 and 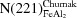 are not the habit planes of these structures. In order to determine the interface plane of the lamellae, the inclination of the colonies was determined on cross sections prepared by FIB. A cross section in Fig. 4 ▸(*a*) is shown in Fig. 4 ▸(*b*). The rotation and inclination with respect to the horizontal image edge and the sample surface, respectively, were determined from Figs. 4 ▸(*a*) and 4 ▸(*b*). These are included in the stereographic projections in Fig. 4 ▸(*c*) as blue lines in the radial and peripheral directions. The intersections of these lines correspond to the interface plane normal. In all investigated cases, 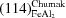 with minor deviations was found as the common plane between FeAl and FeAl_2_. Owing to the distinct orientation relationship, this coincides well with 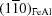. An according low-indexed direction in FeAl_2_ within 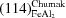 could be identified as 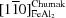. Moreover, 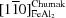 is found to be parallel to 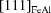. Thus, the orientation relationship between FeAl and FeAl_2_ can now be expressed in terms of the habit planes in the following way: 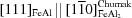 and 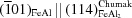. This is discussed on the basis of a crystallographic model in §3.4 in more detail. In addition, there is no evidence for an alternating orientation change or twinning of the FeAl lamellae.

### 3.3. Directionally solidified material   

**Figure 5:**
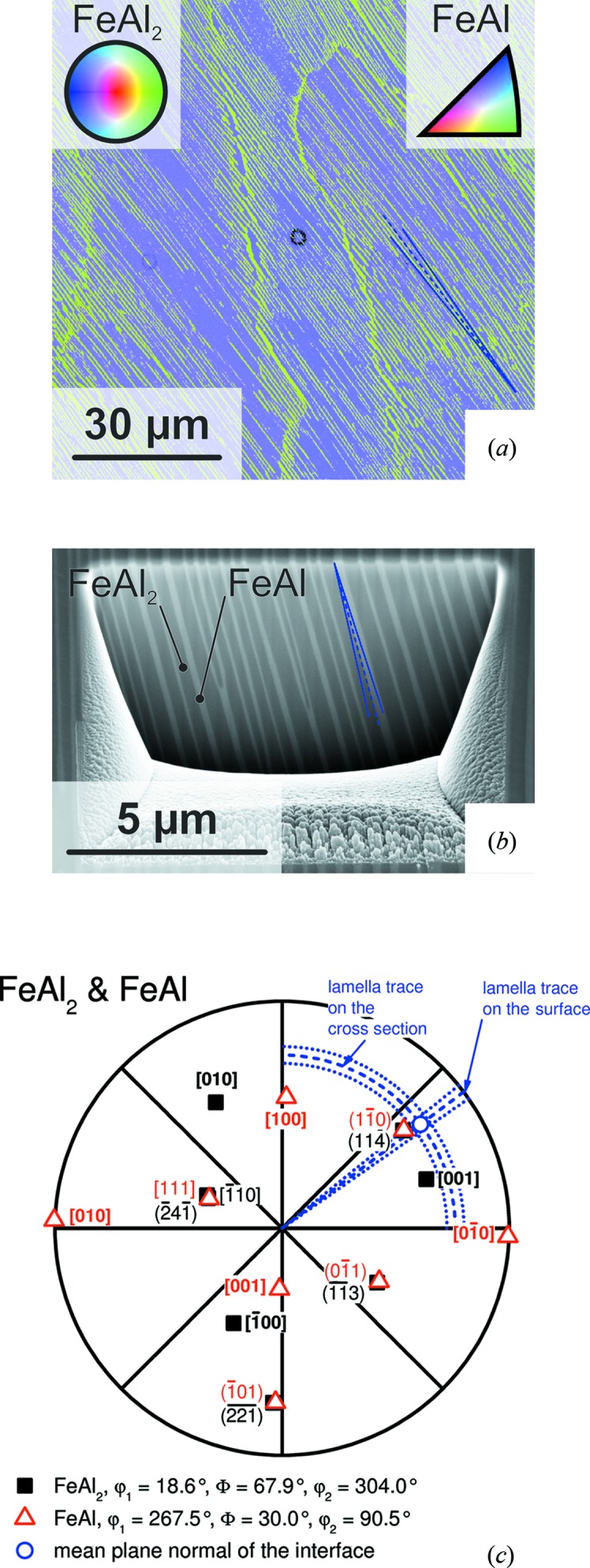
(*a*) Orientation mapping according to the inverse pole figure of the solidification direction on the cross section of the rod (same insets as in Fig. 4 ▸). Missing points in the centre of the image are caused by beam damage. (*b*) An example of a SEM micrograph on a cross section prepared by FIB (secondary electron contrast, tilt-corrected). The rotation and inclination with respect to the horizontal image edge and the sample surface, respectively, are highlighted by blue lines. The mean value obtained by analysing several colonies is indicated by a dashed line – the standard deviation by dotted lines. (*c*) Stereographic projection of the determined orientations of the colony in the centre of (*a*). Blue lines indicate rotation and inclination of the lamella traces according to (*a*) and (*b*). For the pole projection of lattice planes, the normal vector of the planes is used.

An orientation mapping of the directionally solidified material analogous to Fig. 4 ▸ is presented in Fig. 5 ▸(*a*). The volume fractions of FeAl and FeAl_2_ are in agreement with the theoretical calculations with approximately 30 and 70 vol.%, respectively. The solidification direction is perpendicular to the plane of the image. The map is characterized by a rotation angle of about (53 ± 2)° with respect to the horizontal edge (blue dashed lines) of the lamellae on the prepared surface as well as homogeneous crystallographic orientations of FeAl and FeAl_2_ with respect to the solidification direction. Hence, the boundaries between the colonies visible in Fig. 5 ▸(*a*) are small-angle colony boundaries. The inclination of the lamellae into the depth of the investigated material is uniform, too. The inclination angle is about (75 ± 3)° with respect to the sample surface as highlighted by blue dashed lines in Fig. 5 ▸(*b*). The stereographic projection in Fig. 5 ▸(*c*) (centre represents the solidification direction) illustrates the determined orientation relationship of the directionally solidified material which is the same as found for the as-cast state (see Fig. 4 ▸ *c*) and the relationships presented in Fig. 2 ▸. The orientation relationship seems to be robust with respect to different conditions of formation: it is observed not only during precipitation of FeAl (Hirata *et al.*, 2008 ▸) but also during fast and slow cooling of fully lamellar material. Again, no alternating FeAl orientations or twinning of FeAl occurred. The determination of the interface plane normal by the measurement of rotation and inclination of the lamellae also reveals the congruence 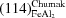 ≡ 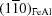 within the interface.

**Figure 6:**
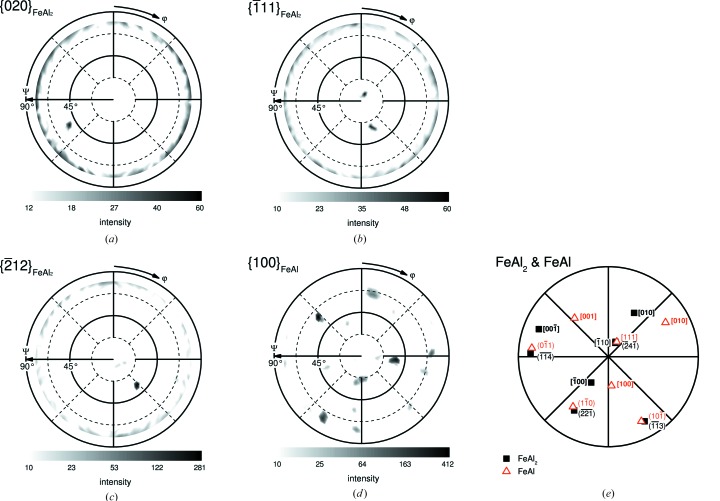
Texture analysis of directionally solidified material: (*a*) to (*d*) experimental XRD pole figures of FeAl_2_ and FeAl (defocusing corrected). (*e*) Stereographic projection of FeAl and FeAl_2_ revealing an alignment of the solidification direction near to the 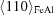 axis of the FeAl phase as well as an orientation relationship according to 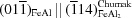 and 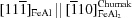. For the pole projection of lattice planes, the normal vector of the planes is used.

In order to verify the local results on a large scale, texture measurements using an X-ray goniometer were carried out. The experimental pole figures are shown in Figs. 6 ▸(*a*)–6 ▸(*d*). Distinct spots within the pole figures indicate strong texturing of the investigated material. In the case of FeAl_2_, the low crystal symmetry causes single spots within the range of the measurement. The slightly increased intensity at high tilt angle visible in Figs. 6 ▸(*a*)–6 ▸(*d*) is caused by insufficient correction of defocusing. Additional signals in the measured pole figures might be caused by overlapping Bragg positions at the chosen 2Θ value. By applying the obtained orientation information, a stereographic projection is calculated and presented in Fig. 6 ▸(*e*) (the simulations of the pole figures are included in the supporting information). FeAl exhibits an alignment of ∼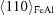 parallel to the solidification direction. As previously shown in the local analysis by EBSD, the global texture measurement provides evidence for the same orientation relationship between FeAl and FeAl_2_.

### 3.4. Crystallographic model   

Based on the experimental determination of the orientation relationship and interface plane between FeAl and FeAl_2_, a model of the crystallographic relationships of the phases participating in the eutectoid decomposition can be derived. As previously pointed out by Stein *et al.* (2010 ▸), the unit-cell parameter of the high-temperature Fe_5_Al_8_ phase is approximately three times larger than that of FeAl. The unit cell of Fe_5_Al_8_ is indeed a 3^3^ superstructure with 52 atoms and two vacancies in comparison to the W prototype. Thus, a cube-on-cube relation is to be expected.

**Figure 7:**
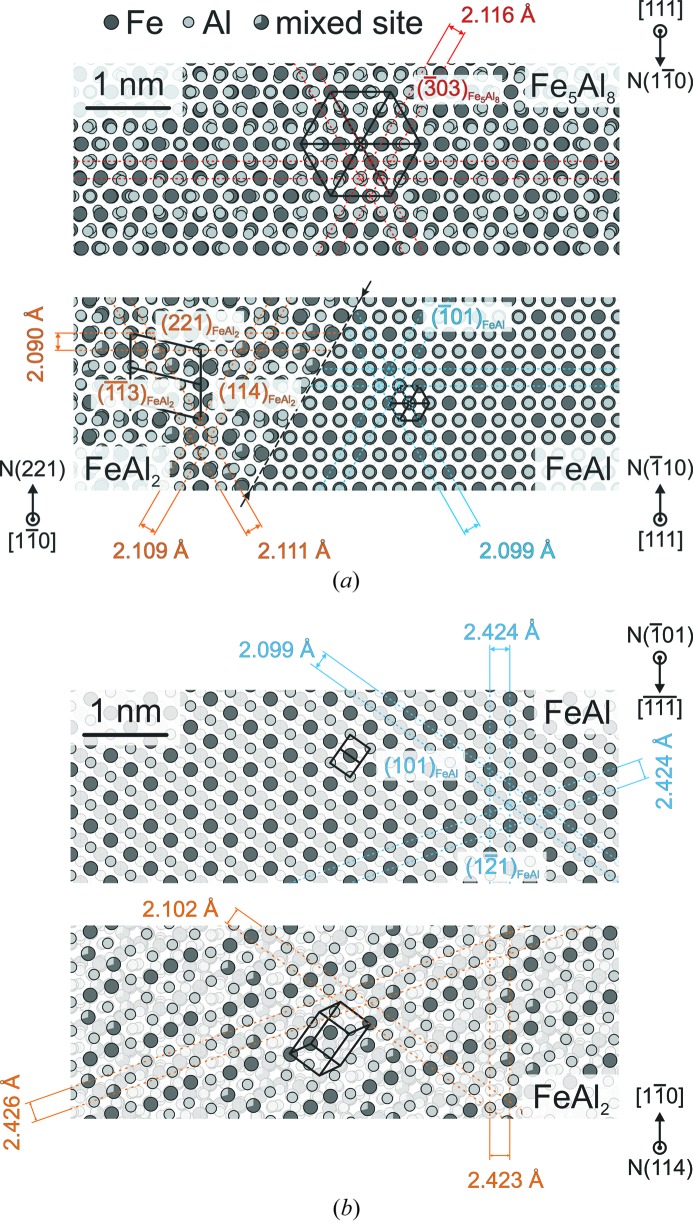
Schematic drawings of the crystallographic model based on the determined orientation relationship and interface of FeAl and FeAl_2_. A possible orientation relationship of Fe_5_Al_8_ is also proposed. (*a*) [111]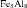 of the high-temperature ∊ phase, 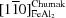 of FeAl_2_ and 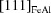 are pointing out-of-plane. The interface planes of FeAl_2_ and FeAl are 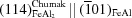. (*b*) Atomic sites within the interface planes 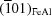 and 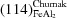. Semi-transparent sites are off the interface plane of the particular phases. The drawings are based on *VESTA* (Momma & Izumi, 2011 ▸) graphics using crystallographic data at 1373 K (Fe_5_Al_8_) and 1353 K (FeAl_2_ and FeAl). The translations between the phases are anticipated or arbitrary. For characterization of the viewing directions, the normal vectors of the lattice planes are used and symbolized by N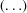.

Fig. 7 ▸ shows the atomic sites based on crystallographic data determined close to the decomposition temperature (Stein *et al.*, 2010 ▸). In Fig. 7 ▸(*a*), the crystal structures of Fe_5_Al_8_ and FeAl are displayed with a [111] viewing direction. The assumption of a cube-on-cube relation seems to be reasonable owing to the common hexagonal arrangement of atomic columns in both phases. According to the determined orientation and interface relationship, FeAl_2_ is viewed from 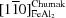 with a 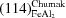 plane towards a 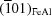 of FeAl. The hexagonal arrangement of atomic columns is observed, too. The hexagonal arrangement is formed by 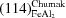, 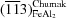 and 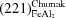 planes (note that 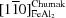 describes the intersection line of all three planes accurately). These planes show the highest structure factors of FeAl_2_ indicating the closest-packed planes within this structure. The highest structure factor and, thus, highest atomic packing is found for 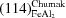. This might result in the exclusive selection of the observed habit planes since 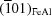 is the closest-packed plane for FeAl. Fig. 7 ▸(*b*) provides a scheme of the atomic sites within the interface between FeAl and FeAl_2_. Thus, 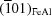 and 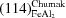 are arranged in-plane whereas the viewing directions of the aforementioned Fig. 7 ▸(*a*) are pointing upwards.

The strains between FeAl and FeAl_2_ which have to be accommodated at the interface can be analysed by the lattice plane distances provided in Fig. 7 ▸(*b*). At the maximum a deviation of 0.14% is observed within the interface, indicating a very good compatibility.

Future local high-resolution investigations with respect to lattice structure and chemistry of the interface should be performed in order to reveal the interface structure as well as the possibility of slip transfer between the investigated phases. 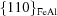 surfaces are known to reorganize to FeAl_2_ commensurate structures for energetic reasons (Baddorf & Chandavarkar, 1996 ▸). Hence, similar effects are also expected for the interface between FeAl and FeAl_2_. This might be facilitated by the changing Al content in both phases during cooling which will lead to locally changing lattice parameters in the vicinity of the interface. In addition, the interface structure of branched lamellae, as shown in Fig. 4 ▸(*b*) and frequently found in this system, remains unclear.

## 4. Conclusions   

This study provides the following main results regarding the orientation and interface relationship of FeAl and FeAl_2_ resulting from a eutectoid decomposition of Fe_5_Al_8_:

(i) The orientation relationship determined by Bastin *et al.* and Hirata *et al.* was confirmed in fully lamellar as-cast and directionally solidified material.

(ii) In addition, the interface of FeAl and FeAl_2_ lamellae is found to be 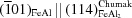. Based on this habit plane the orientation relationship can be set up by 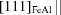 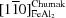.

(iii) There is no evidence for an alternating orientation change of FeAl lamellae suggesting that FeAl_2_ has inversion symmetry.

(iv) The directional solidification leads to a near 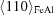 || solidification direction texture.

(v) The observed orientation and interface relationship can be described well by a crystallographic model. In addition, a possible orientation relationship to the high-temperature Fe_5_Al_8_ phase is proposed.

## Supplementary Material

Orientation mapping according to the inverse pole figure of the surface normal on a cross section of the arc-melted button (see insets; center of the IPF of FeAl2 corresponds to [001]_FeAl2^Chumak). Blue lines indicate minima and maxima of the lamella trace rotation with respect to the horizontal edge in the different parts of the image. Stereographic projections of the determined orientations of the colonies A to D. Blue lines indicate rotation and inclination of the lamella traces.. DOI: 10.1107/S1600576716000911/kc5028sup1.pdf

Texture analysis of directionally solidified material: experimental XRD pole figures of FeAl2 and FeAl (defocusing corrected). Simulation of the according poles observed in the experimental pole figures. The center of the pole figure corresponds to the solidification direction. Filled symbols represent poles which are actually used in order to match the orientation of the unit cells.. DOI: 10.1107/S1600576716000911/kc5028sup2.pdf

## Figures and Tables

**Table 1:** Comparison of lattice parameters of FeAl_2_ according to the relevant references and appropriate transformations

Reference/transformation	*a* (Å)	*b* (Å)	*c* (Å)	α (°)	β (°)	γ (°)	Volume (Å^3^)
Chumak *et al.* (2010 ▸)	4.8745	6.4545	8.7361	87.93	74.396	83.062	262.79
Corby & Black (1973 ▸)	4.878	6.461	8.800	91.75	73.27	96.89	263.69
Transformation by **a**′ = **a**, **b**′ = −**b**, **c**′ = **a** − **c**	4.878	6.461	8.748	87.93	74.45	83.11	263.69
Bastin *et al.* (1978 ▸)	7.594	16.886	4.862	89.55	122.62	90.43	525.10
Transformation by **a**′ = **c**, **b**′ = **a** + **c**, **c**′ = ½ (**b** + **c**)	4.862	6.442	8.806	88.24	73.48	83.15	262.55

## Footnotes

1 Throughout the article, superscripts in crystallographic notations highlight the crystallographic data set and subscripts denote the phase. Moreover, […] and 〈…〉 are used for directions and crystallographically equivalent directions, respectively, whereas (…) and {…} are used for planes and crystallographically equivalent planes, respectively. The mathematical expression describing two parallel planes in the two structures (…)_FeAl_ || (…)_FeAl_2__ holds true also for the plane normals of the mentioned planes. If directions are parallel to plane normals, the description by Hirata *et al.* in the form of […]_FeAl_ || N(…)_FeAl_2__ is used.
