# Supplementary material for: Orientation relationship of eutectoid FeAl and FeAl2
Source: J Appl Crystallogr. 2016 Feb 24;49(Pt 2):442–9. doi: 10.1107/S1600576716000911 (PMC4815872; doi:10.1107/S1600576716000911)
Supplement: Supplementary file 1 [file j-49-00442-sup1.pdf]

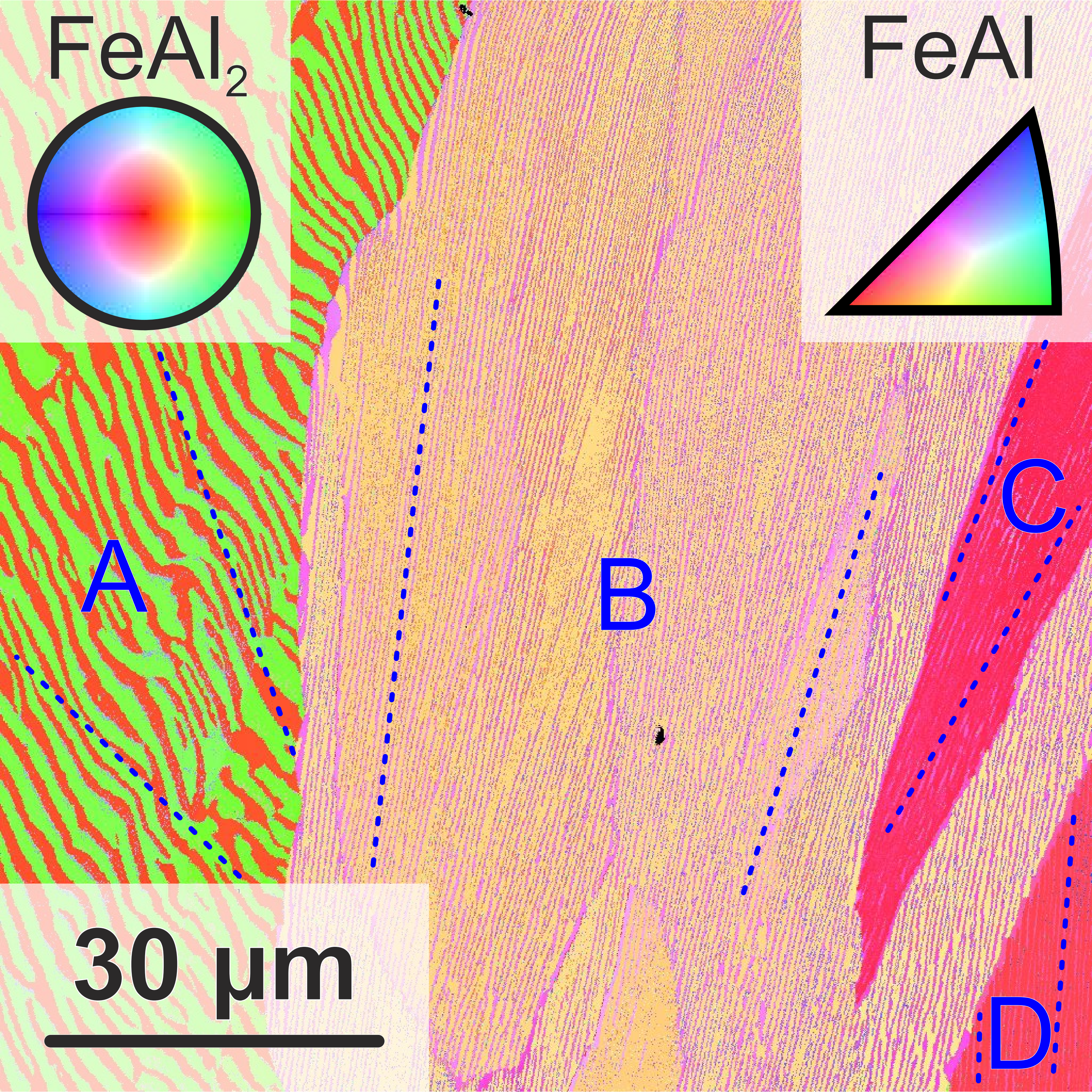

$FeAl_2$
FeAl
A
B
C
D
30 µm

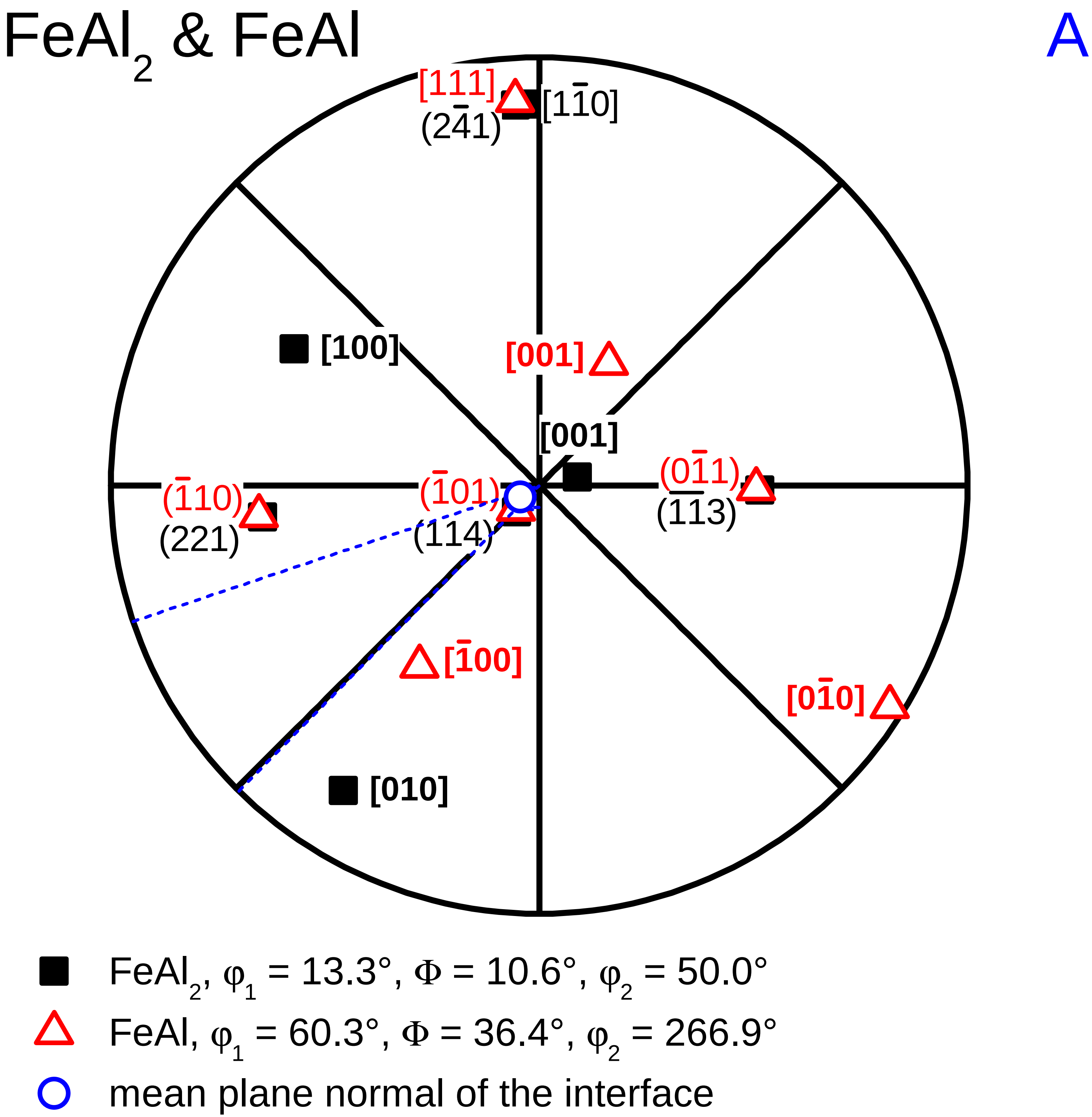

FeAl2 & FeAl
A
[111]
(2$\bar{4}$1)
[1$\bar{1}$0]
[100]
[001]
[001]
($\bar{1}$10)
(221)
($\bar{1}$01)
(114)
(0$\bar{1}$1)
($\bar{1}\bar{1}$3)
[$\bar{1}$00]
[0$\bar{1}$0]
[010]
FeAl2, φ1 = 13.3°, Φ = 10.6°, φ2 = 50.0°
FeAl, φ1 = 60.3°, Φ = 36.4°, φ2 = 266.9°
mean plane normal of the interface

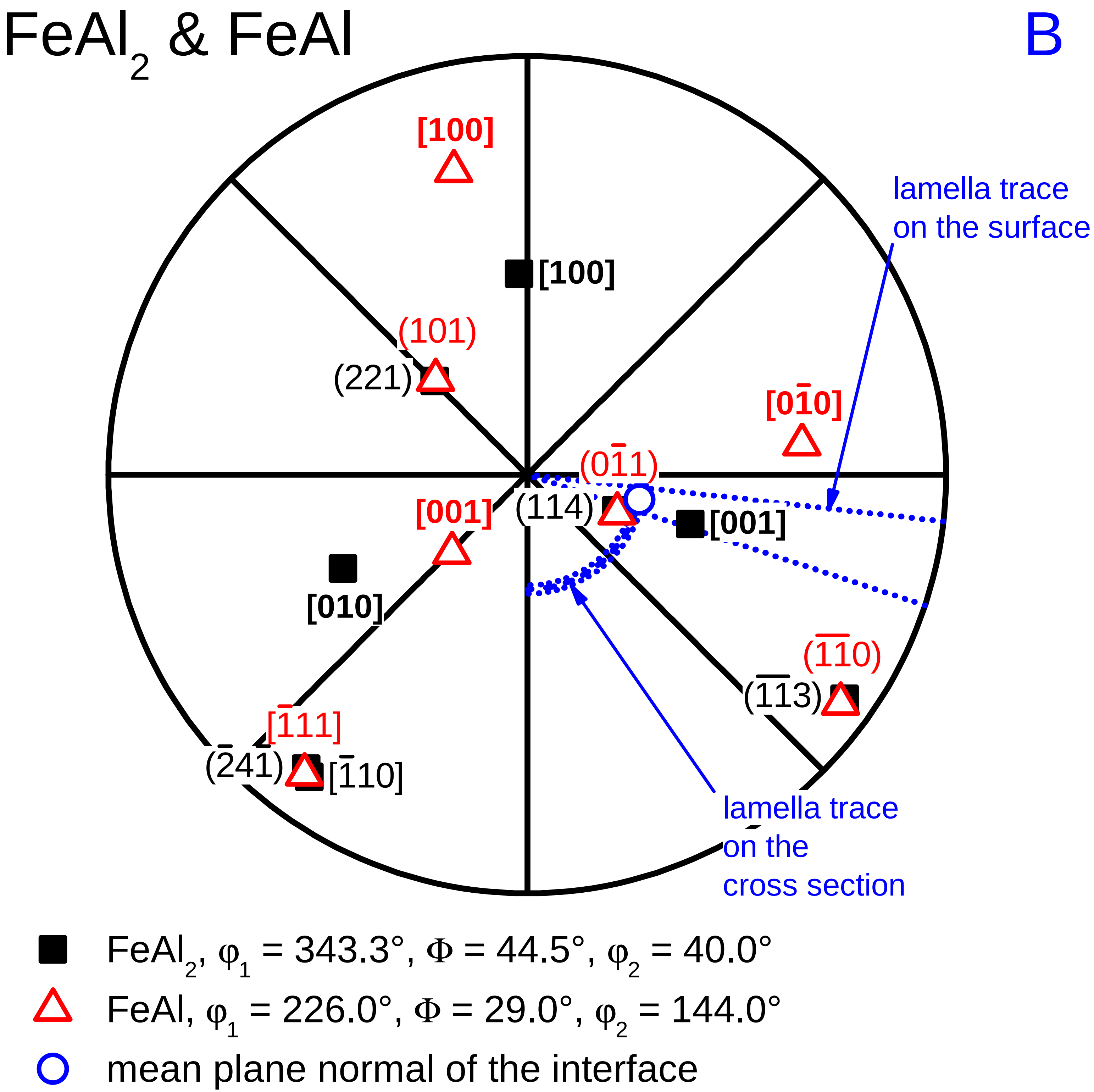

FeAl$_2$ & FeAl
B
[100]
[100]
(101)
(221)
[0$\bar{1}$0]
lamella trace
on the surface
(0$\bar{1}$1)
(114)
[001]
[001]
[010]
($\bar{1}\bar{1}$0)
($\bar{1}\bar{1}$3)
[$\bar{1}$11]
($\bar{2}$4$\bar{1}$)
[$\bar{1}$10]
lamella trace
on the
cross section
FeAl$_2$, φ_1 = 343.3°, Φ = 44.5°, φ_2 = 40.0°
FeAl, φ_1 = 226.0°, Φ = 29.0°, φ_2 = 144.0°
mean plane normal of the interface

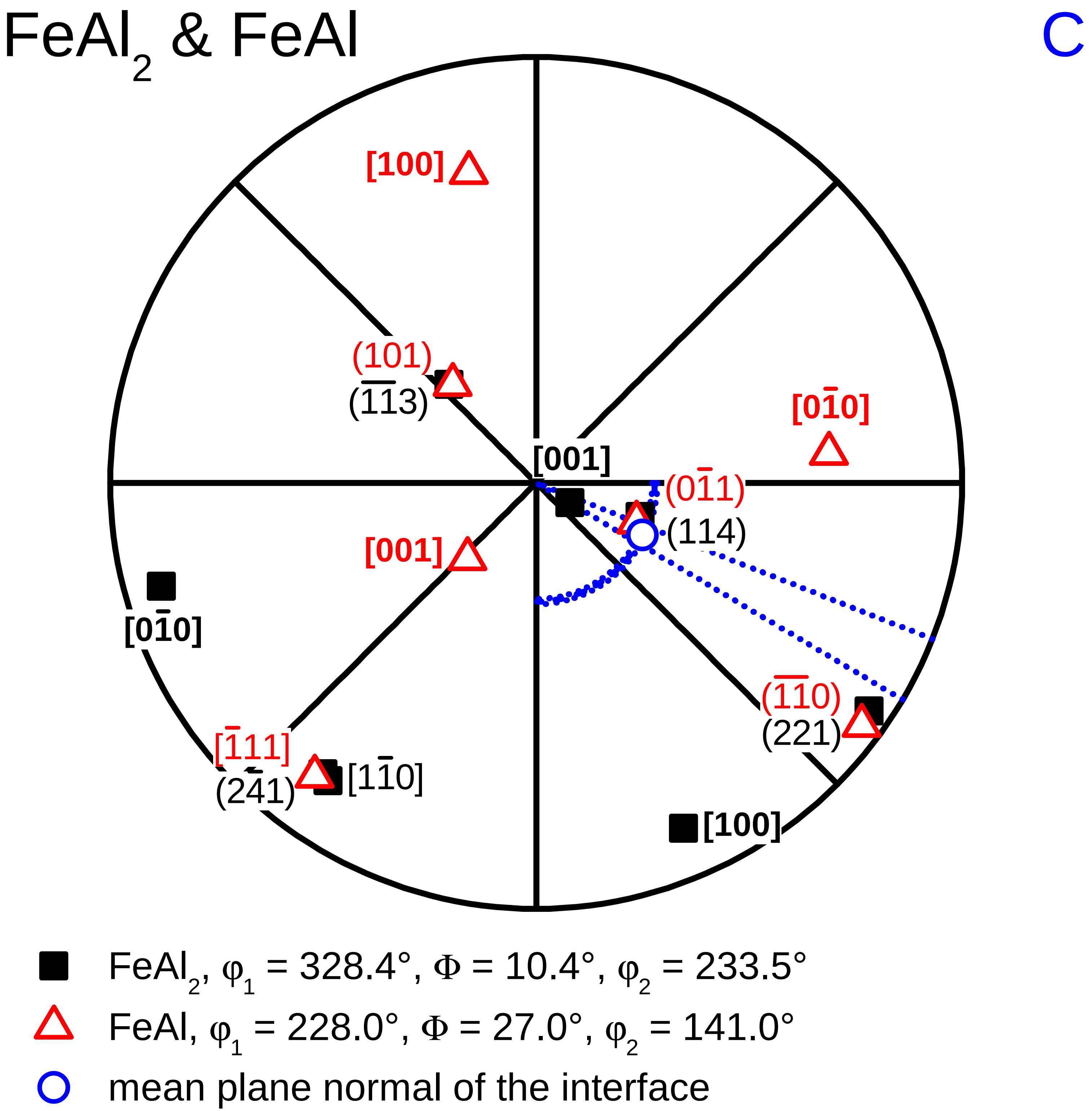

$FeAl_2$ & FeAl
C
[100]
(101)
$(\bar{1}\bar{1}3)$
$[0\bar{1}0]$
[001]
$(0\bar{1}1)$
(114)
[001]
$[0\bar{1}0]$
$(\bar{1}\bar{1}0)$
(221)
$[\bar{1}11]$
$(2\bar{4}1)$
$[1\bar{1}0]$
[100]
$FeAl_2$, φ_1 = 328.4°, Φ = 10.4°, φ_2 = 233.5°
FeAl, φ_1 = 228.0°, Φ = 27.0°, φ_2 = 141.0°
mean plane normal of the interface

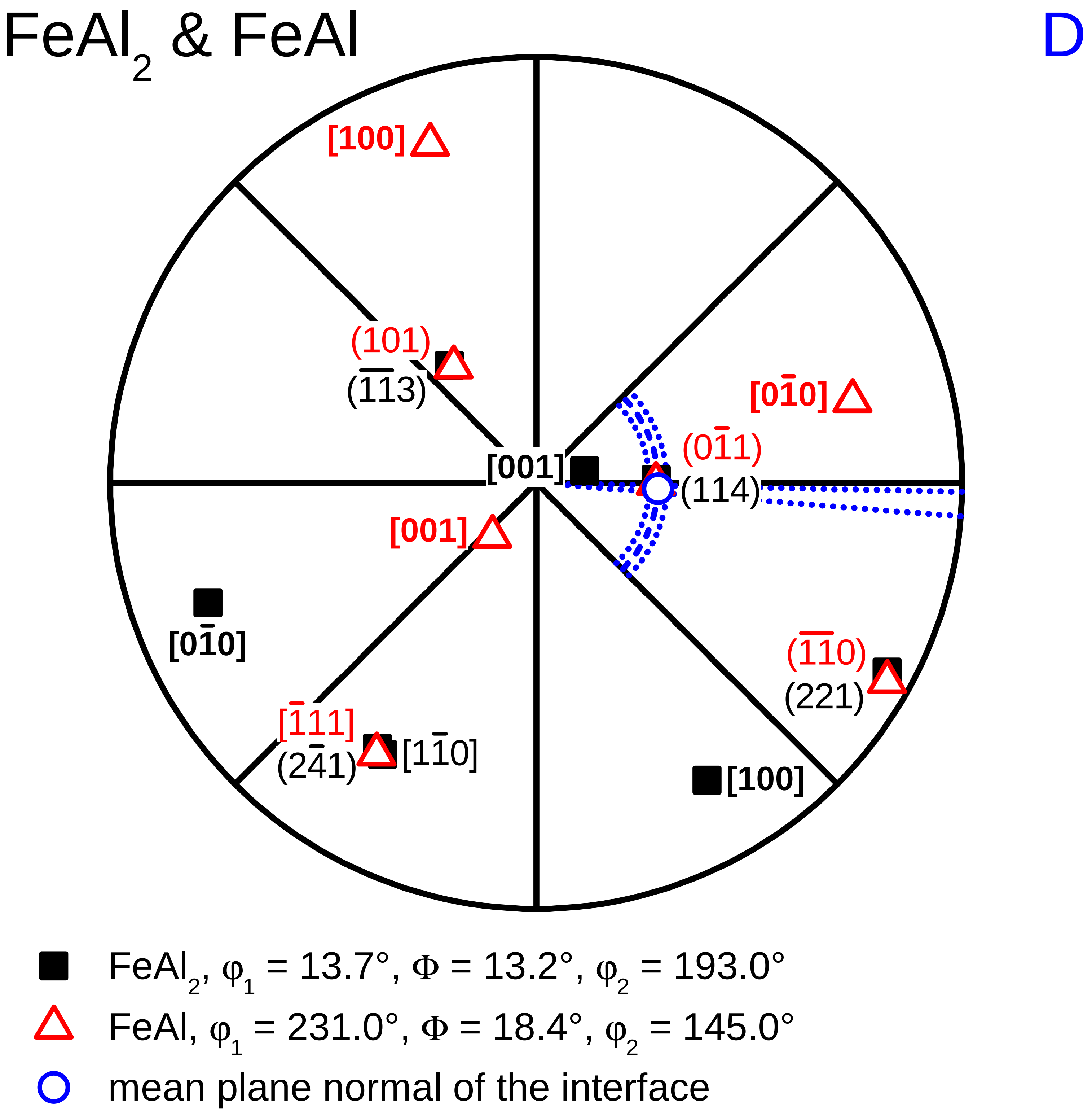
$FeAl_2$ & FeAl
D
[100]
(101)
($\bar{1}\bar{1}3$)
[0$\bar{1}$0]
(0$\bar{1}$1)
[001]
(114)
[001]
[0$\bar{1}$0]
($\bar{1}\bar{1}$0)
(221)
[$\bar{1}$11]
(2$\bar{4}$1)
[1$\bar{1}$0]
[100]
$FeAl_2$, φ_1 = 13.7°, Φ = 13.2°, φ_2 = 193.0°
FeAl, φ_1 = 231.0°, Φ = 18.4°, φ_2 = 145.0°
mean plane normal of the interface
