# Supplementary material for: Orientation relationship of eutectoid FeAl and FeAl2
Source: J Appl Crystallogr. 2016 Feb 24;49(Pt 2):442–9. doi: 10.1107/S1600576716000911 (PMC4815872; doi:10.1107/S1600576716000911)
Supplement: Supplementary file 2 [file j-49-00442-sup2.pdf]

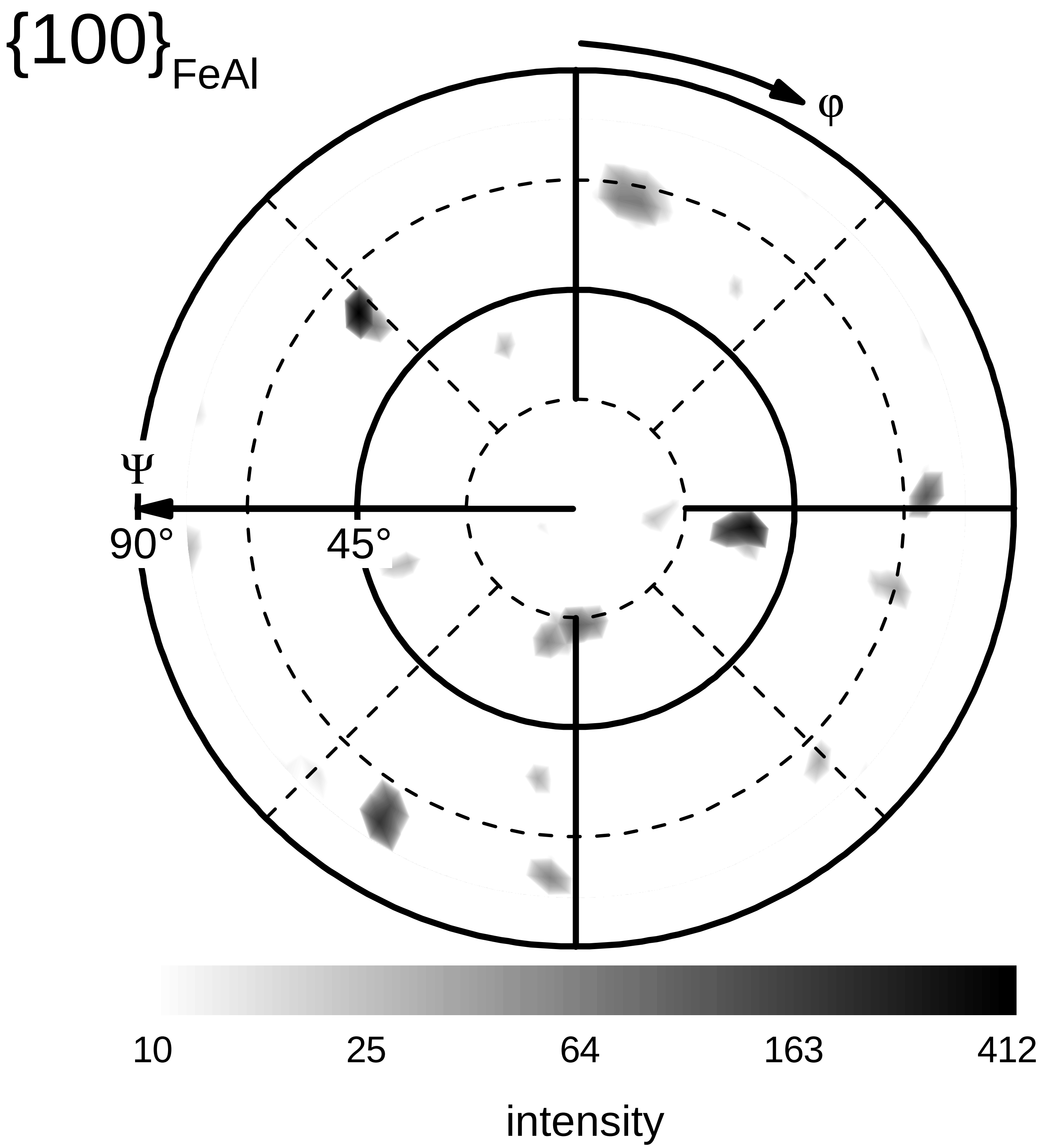
{100}FeAl
φ
Ψ
90°
45°
10
25
64
163
412
intensity

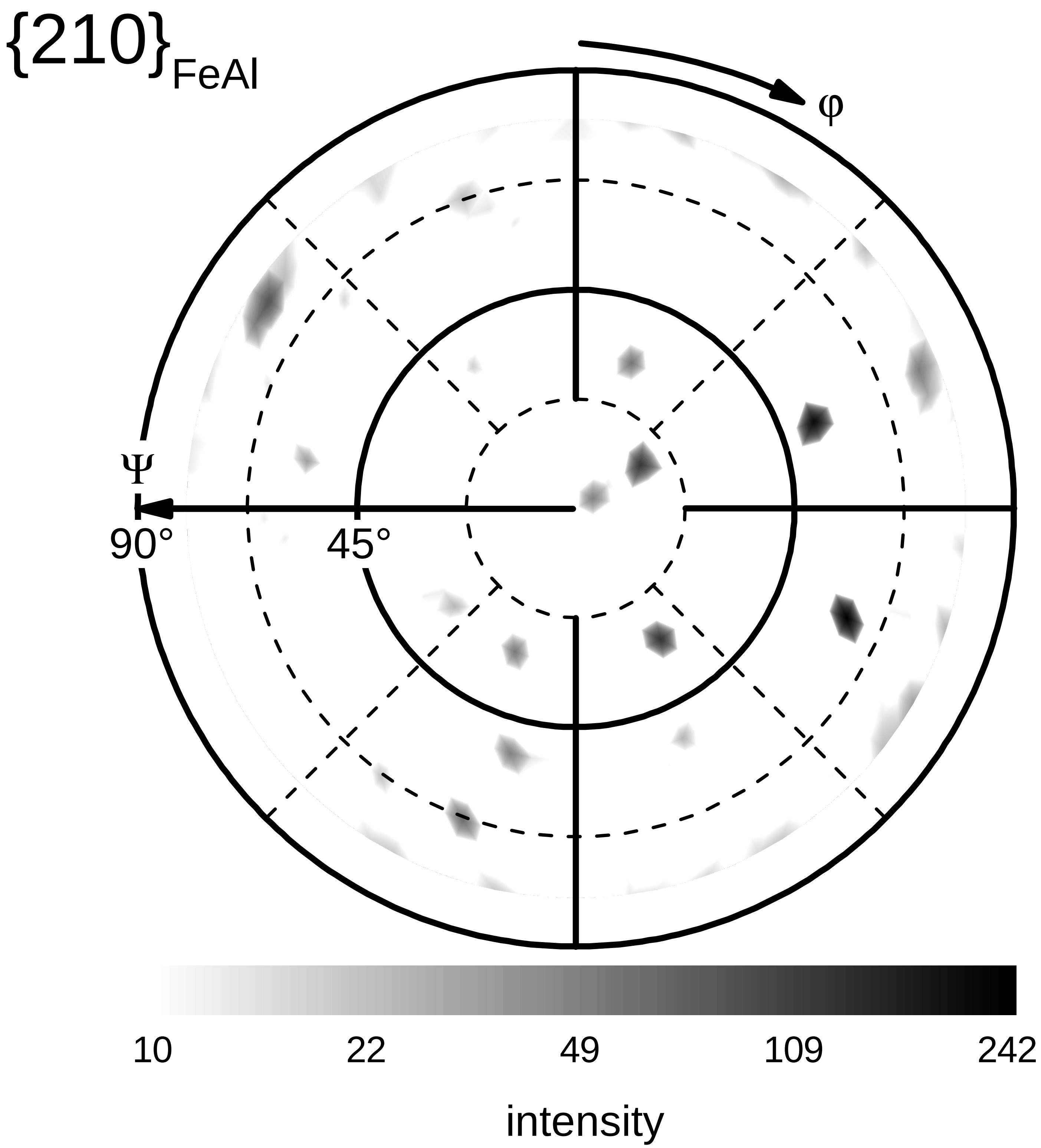

{210}FeAl
φ
Ψ
90°
45°
10
22
49
109
242
intensity

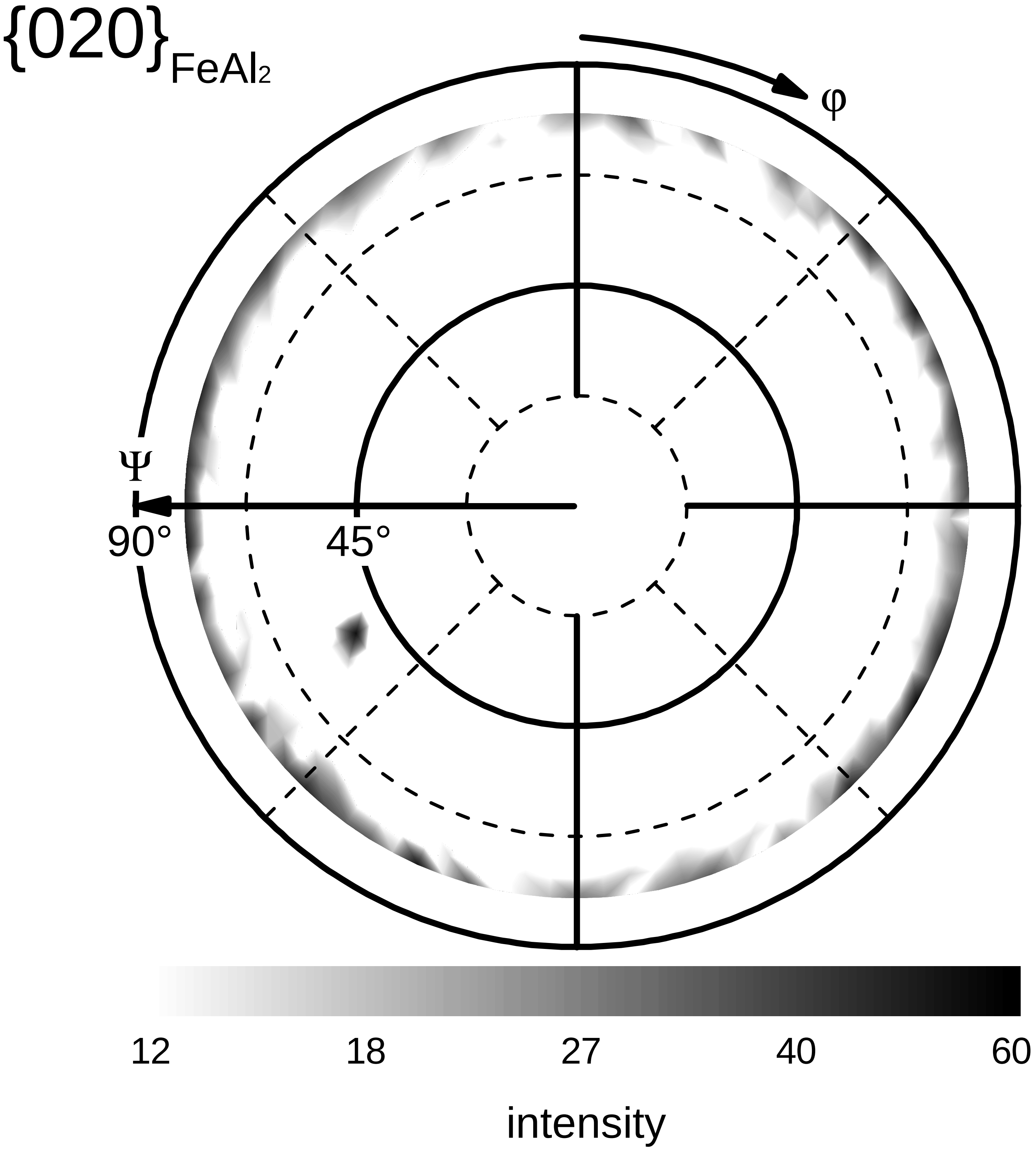

{020}FeAl2
φ
Ψ
90°
45°
12
18
27
40
60
intensity

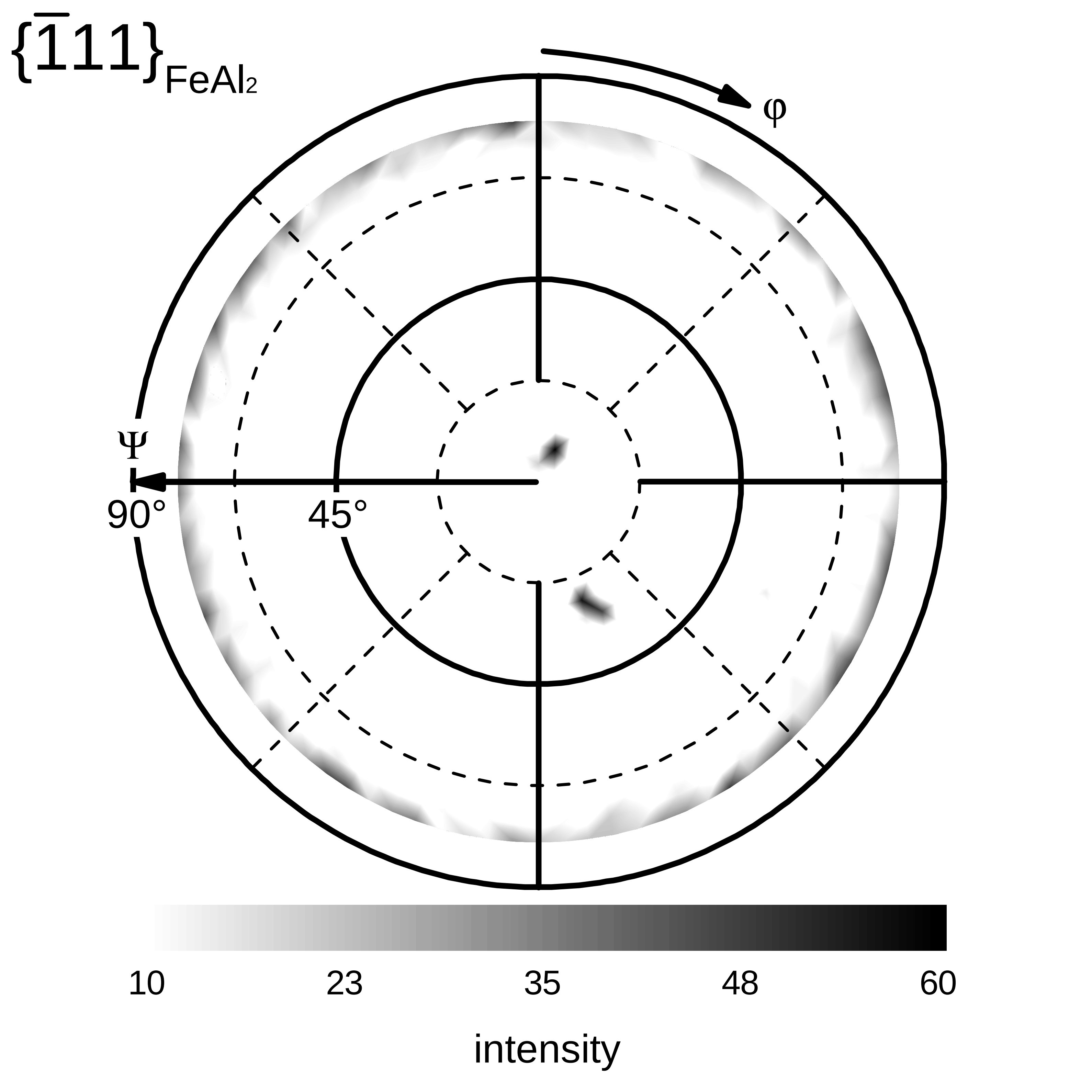
{$\bar{1}$11}$_{FeAl_2}$
φ
Ψ
90°
45°
10
23
35
48
60
intensity

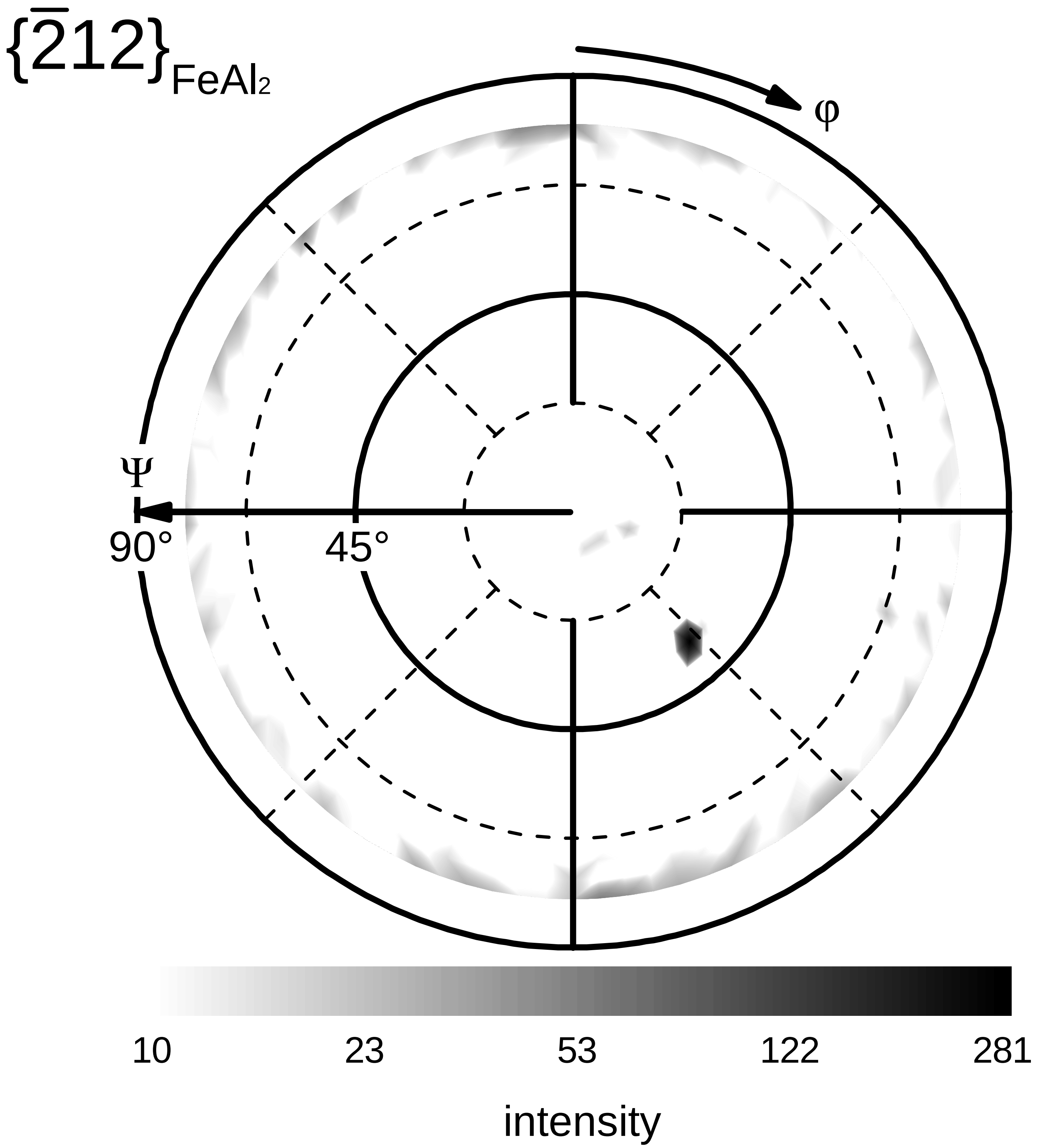
{$\bar{2}$12}FeAl2
φ
Ψ
90°
45°
10
23
53
122
281
intensity

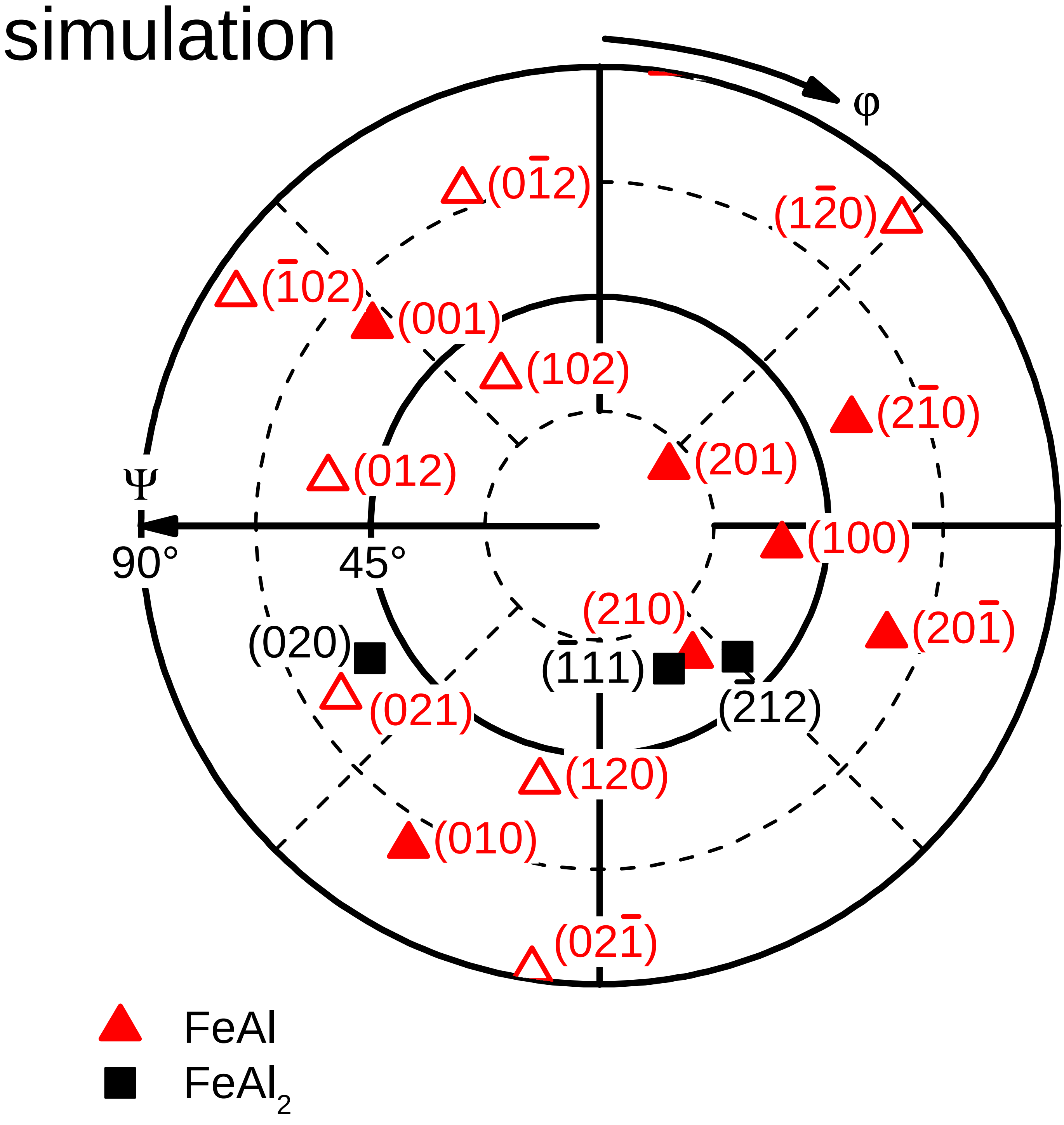

simulation
φ
Ψ
90°
45°
(01̄2)
(12̄0)
(1̄02)
(001)
(102)
(21̄0)
(012)
(201)
(100)
(210)
(201̄)
(020)
(1̄11)
(212)
(021)
(120)
(010)
(021̄)
FeAl
$FeAl_2$

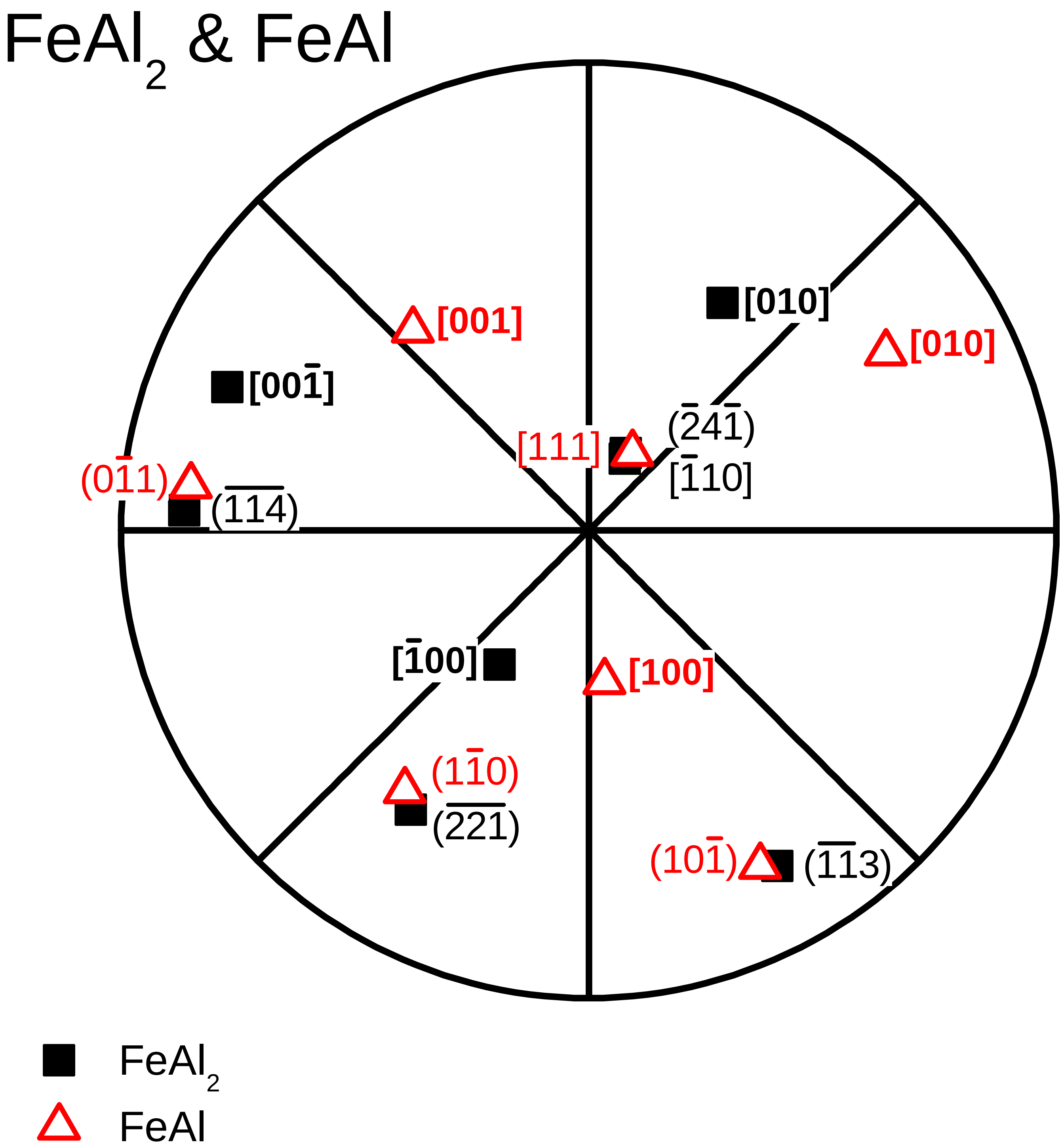

FeAl2 & FeAl
[010]
[010]
[001]
[001̄]
(2̄41̄)
[111]
[1̄10]
(01̄1)
(1̄1̄4̄)
[1̄00]
[100]
(11̄0)
(2̄2̄1̄)
(101̄)
(1̄1̄3)
FeAl2
FeAl
